# Paradigm Shift Toward Digital Neuropsychology and High-Dimensional Neuropsychological Assessments: Review

**DOI:** 10.2196/23777

**Published:** 2020-12-16

**Authors:** Thomas Parsons, Tyler Duffield

**Affiliations:** 1 Computational Neuropsychology & Simulation University of North Texas Denton, TX United States; 2 Oregon Health & Science University Portland, OR United States

**Keywords:** neuropsychology, technology, informatics, machine learning, big data, virtual reality, smartphone, mobile phone

## Abstract

Neuropsychologists in the digital age have increasing access to emerging technologies. The National Institutes of Health (NIH) initiatives for behavioral and social sciences have emphasized these developing scientific and technological potentials (eg, novel sensors) for augmented characterization of neurocognitive, behavioral, affective, and social processes. Perhaps these innovative technologies will lead to a paradigm shift from disintegrated and data-poor behavioral science to cohesive and data-rich science that permits improved translation from bench to bedside. The 4 main advances influencing the scientific priorities of a recent NIH Office of Behavioral and Social Sciences Research strategic plan include the following: integration of neuroscience into behavioral and social sciences, transformational advances in measurement science, digital intervention platforms, and large-scale population cohorts and data integration. This paper reviews these opportunities for novel brain-behavior characterizations. Emphasis is placed on the increasing concern of neuropsychology with these topics and the need for development in these areas to maintain relevance as a scientific discipline and advance scientific developments. Furthermore, the effects of such advancements necessitate discussion and modification of training as well as ethical and legal mandates for neuropsychological research and praxes.

## Introduction

Clinical neuropsychologists have traditionally developed and validated parsimonious assessment tools using basic technologies (ie, pencil and paper protocols, general linear model). Advances have predominantly occurred in expanded normative standards throughout the history of this profession [1]. Although these low-dimensional tools are well-validated assessments of basic cognitive constructs, they have limited presentation (static 2D stimuli) and logging capabilities (which require manual logging of responses). Moreover, low-dimensional approaches limit their statistical modeling (typically linear) to combinations of features relative to a set of weights for predicting the value of criterion variables. Some neuropsychologists may argue that the parsimony offered by low-dimensional tools reflects the reality of a much higher-dimensional deficit. However, low-dimensional tools may offer diminished interpretations of complex phenomena.

**Figure 1:**
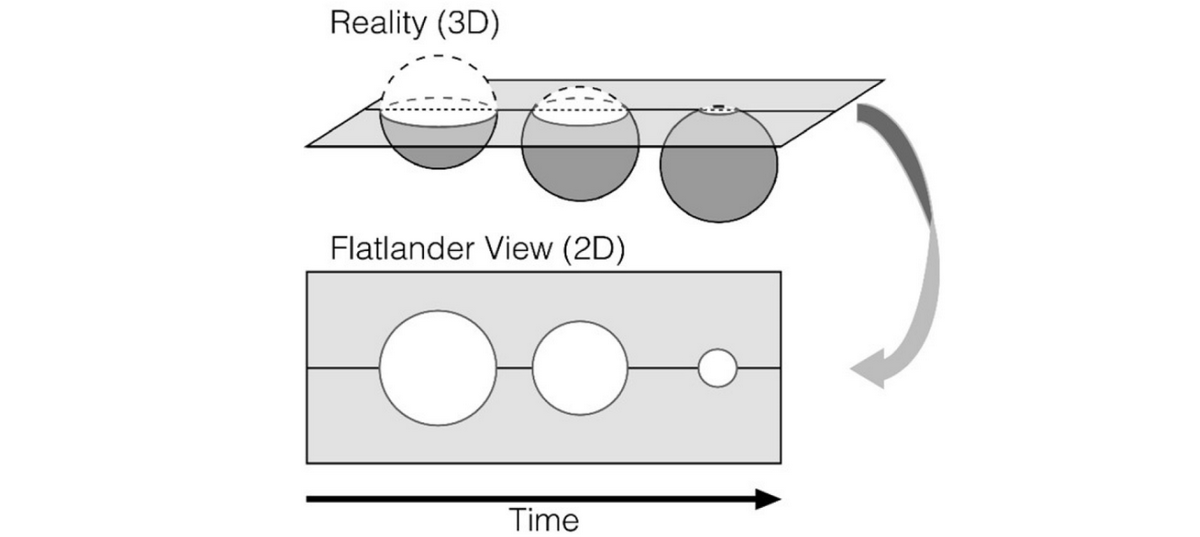
A Square cannot perceive his world as anything other than 2-dimensional. Reprinted with permission.

The preference for low-dimensional tools is apparent in surveys of assessments used by neuropsychologists [2,3]. This conservativism has resulted in neuropsychological assessments that have hardly changed since the original scales were established in the early 1900s [4,5]. Low-dimensional neuropsychological assessment tools place the neuropsychologist on par with the 19th century literary work on the nature of perception and dimensionality. Specifically, the narrator, A Square, resides in Flatland with residents (Flatlanders) whose perception is limited to 2 dimensions. After a discussion with a *Stranger* (a sphere), A Square comes to understand how complex and high dimensional the world is. Unfortunately, A Square is jailed for holding and communicating heretical beliefs [6]. For neuropsychologists, low-dimensional technologies have led us to search for simplified explanations of complex phenomena, which limits our ability to develop, validate, interpret, and communicate useful models of human neuropsychology. Recently, cognitive psychologists have called this the *Flatland fallacy*. They contend that the Flatland fallacy can be surmounted by formalizing psychological theories as computational models that have the capacity to make precise predictions about cognition and/or behavior (Figure 1) [7].

These authors explain that in the limited perspective of the Flatlanders’ view (bottom of the figure), a 3D object (sphere) seems to be fluctuating magnitudes (an expanding and reducing circle). However, the reality is that (top of the figure) this object is merely progressing through a lower‐dimensional plane. The low-dimensional perspective leads to a false understanding of reality. Similarly, neuropsychologists may incorrectly determine that the low level of dimensionality correctly describes neuropsychological or psychological phenomena. In fact, they may be missing the complexity and high dimensionality of neuropsychological phenomena.

Cognitive psychologists also contend that unitary cognitive constructs such as *attention* are limited and prevent psychologists from deepening the understanding of complex, or high-dimensional, phenomena. First, theories of unitary cognitive constructs are based on circular reasoning. Complex phenomena such as the conception of *attention* are explained by presumptive attentional systems. Instead, psychologists should model the parallel, reciprocal, and iterative interactions between context and neural or functional processes. This would enhance the characterization of physically executed actions [8]. Similarly, the analytical approach to psychology is problematic because (1) an exhaustive definition is proposed (eg, attention), (2) assumed subfunctions are identified (eg, selective, sustained, or divided attention) with separable functional and neuronal processes (or dedicated systems), and (3) research concentrates on specific tasks that purport to measure the theoretical subfunctions rather than underlying processes required to execute an efficient behavior in a particular situation [9]. Commonalities between subfunctions and other constructs (eg, working memory) are often not empirically distinguishable and by no means imply that the underlying functional and neural processes are different or separable. These authors propose that rather than being rigidly adherent to prior cognitive conceptual frameworks, psychologists should model mechanisms and processes (sensory, motor, and cognitive) that are found in several complex behaviors. These behaviors may run in parallel or interact across stimulus properties, time, and goals to achieve an outcome.

How do neuropsychologists move from low-dimensional neuropsychology to high-dimensional neuropsychology? The National Institutes of Health (NIH) offers initiatives for neuropsychologists interested in higher-dimensional tools, including (1) integrating neuroscience into behavioral and social sciences, (2) transformative advances in measurement science, (3) digital intervention platforms, and (4) large-scale population cohorts and data integration [10]. Similarly, the NIH Brain Research through Advancing Innovative Neurotechnologies (BRAIN) initiative seeks high-dimensional approaches to understand brain disorders (eg, Alzheimer disease, Parkinson disease, depression, and traumatic brain injury) and accelerating the technologies for high-dimensional modeling of how the brain records, processes, uses, stores, and retrieves vast quantities of information [11]. Neuropsychologists can enhance work conducted in NIH initiatives by offering interpretations of neuroscience findings based on clinical expertise.

After a brief consideration of the historic progression of neuropsychological assessment technologies, there is an elucidation of current NIH initiatives for the behavioral and social sciences as well as evaluations of current neuropsychological assessment technologies.

## A Very Brief History of Neuropsychological Assessment Technologies

Neuropsychology has experienced a number of advances as it developed from a primarily qualitative practice to a more objective and evidence-based approach [12], with expanded normative standards [1], performance validity testing [13], and cross-cultural considerations [14]. Although these improvements have aided the investigation of neurocognitive functions, there are increasing discussions on the need to enhance the dimensionality of neuropsychological assessments and computational modeling [1,4,5,15-22].

The technological and theoretical development of neuropsychological assessment can be understood in terms of dimensional waves of technological adoption [5]. In Neuropsychology 1.0, neuropsychological assessments accentuate the development of low-dimensional and construct-driven (ie, simple stimulus presentations of stimuli to test abstract concepts like working memory) paper-and-pencil measures. In Neuropsychology 2.0, there is a technological move to automated administration, scoring, and in some instances the interpretation of low-dimensional stimulus presentations using computerized approaches (eg, NIH Toolbox and video teleconferencing) [23-26]. Concurrently, technological developments in neuroimaging have changed the role of neuropsychological assessments, from lesion localization to predictions about a patient’s ability to perform activities of daily living. Finally, Neuropsychology 3.0 reflects contemporary advances in high-dimensional (dynamic and ecologically valid simulation, logging, and modeling of everyday activities) tools.

Some neuropsychologists are hesitant to move from low-dimensional to high-dimensional tools because computerized assessments may introduce errors and/or decrease the reliability of the assessment process by means of automation [27]. Although there have been improvements in computational power and security, developers of high-dimensional technologies need to take appropriate actions to ensure proper implementation [28]. Furthermore, normative efforts are ongoing for high-dimensional assessments, and continued validation of advanced platforms and novel data analytic approaches is needed.

Three decades ago, clinical psychologists were urged to adopt progressively available advanced technologies [29]. Concurrently, in the 1980s, neuropsychologists started discussing the potential of computerized neuropsychological assessments [30]. It was subsequently pointed out that when compared with progress in our everyday technologies, psychological assessments had barely progressed throughout the 20th century (eg, Wechsler scales) [31]. Technologies found in neuropsychological testing can be compared with now obsolete black-and-white televisions, vinyl records, rotary-dial telephones, and the first commercial computer made in the United States (ie, Universal Automatic Computer I). Assessment technologies need to progress in ideas, not just new measures [31].

In the late 1990s, it was discussed how neuropsychology lagged behind (in absolute terms and in comparison with) other clinical neurosciences. Clinical neuropsychologists continued to use many of the same tools that have been developed decades earlier. Moreover, new tests that were coming out were not conceptually or substantively better than the ones from decades earlier (eg, Wechsler scales). Dodrill [32] pointed to the fact that in the 1970s, there was little difference in the technological progress of neurology and neuropsychology. This changed with the advent of computerized tomographic scanning, and neuropsychologists were no longer consulted for lesion localization. Neuroimaging advances allowed neurologists to better understand and treat neurologic pathophysiology [33]. Dodrill [32] suggests that if technological developments in neurology had been as slow as that found in neuropsychology, then neurologists would be limited to pneumoencephalograms and radioisotope scans. These procedures are deemed primeval by current neuroradiological standards.

**Figure 2:**
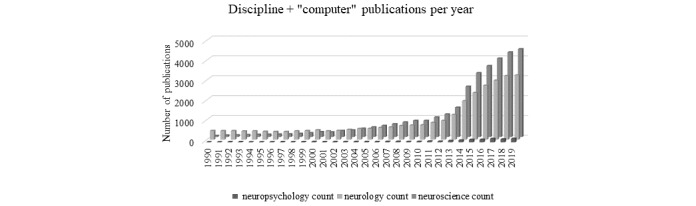
Proliferation of publications identified in the PubMed database over time. Search terms: “computer” by discipline (eg, “neuropsychology,” “neurology,” “neuroscience”).

**Figure 3:**
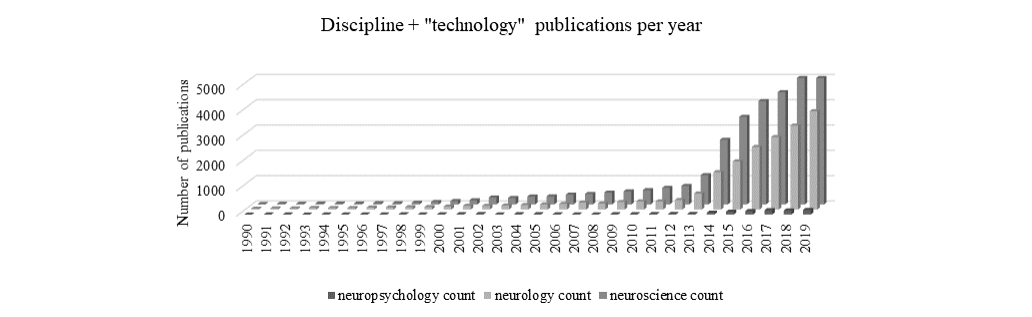
Proliferation of publications identified in the PubMed database over time. Search terms: “technology” by discipline (eg, “neuropsychology,” “neurology,” “neuroscience”).

**Figure 4:**
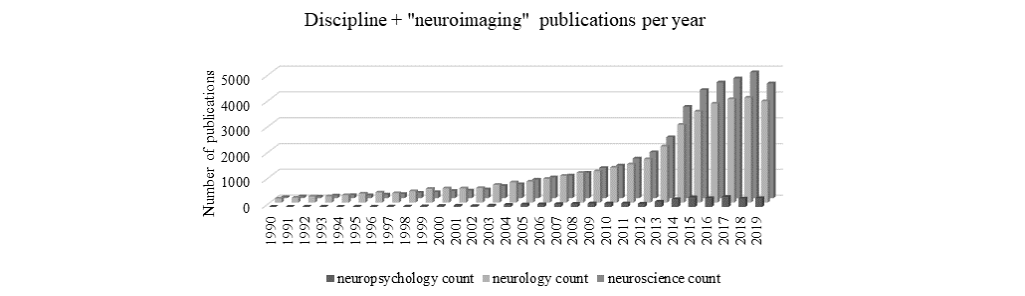
Proliferation of publications identified in the PubMed database over time. Search terms: “neuroimaging” by discipline (eg, “neuropsychology,” “neurology,” “neuroscience”).

To get an idea of where neuropsychology is today, basic searches were performed on July 29, 2020, to tally the number of technology publications per discipline. The first search included a PubMed search with the search terms “computer” AND (“neuropsychology” OR “neurology” OR “neuroscience”) from 1990 to 2019 (Figure 2). A second and third search using the terms “technology” and “neuroimaging” instead of “computer” revealed similar findings (Figures 3 and 4, respectively). Figures 2-4 show the number of publications by year that resulted from each of these 3 broad literature searches.

Findings from these basic searches suggest that high-dimensional technologies have vastly greater representations in neurology and the neurosciences. The inclusion of technologies is very recently increasing in neuropsychology but is explicitly not keeping pace with other neurosciences. Similarly, a survey of rates of neuropsychologists using computerized instruments revealed that only 6% of the 693 neuropsychology assessments were computerized [3]. The average respondent reported that they rarely used computerized tests. An increased likelihood of computerized assessment use was apparent for early career neuropsychologists.

## NIH’s Transformative Opportunities for the Behavioral and Social Sciences

### Integrating Neuroscience Advances Into Clinical Neuropsychology

High-dimensional technologies such as functional neuroimaging provide real-time observations of brain function that challenge the validity of some low-dimensional paper-and-pencil technologies. Impairments following brain injury are rarely a single type of processing, and there is no one-to-one relationship between neuropsychological functions and brain structures and systems. Similar symptoms can arise from various injury types, and the same underlying injury can result in a variety of different symptoms. Although the integration of neuroimaging and neuropsychological methods has improved our understanding of brain functions, specific neuropsychological functions are typically associated with activation in multiple brain regions (distributed processing). Advances in methods and high-dimensional technologies offer promise for redefining previous understandings of cognitive functions (eg, elucidation of multiple types of processing speed) and introduction of novel (and complex and dynamic) cognitive functions [34].

Neuropsychologists are increasingly arguing for neuropsychological models established in terms of patients’ reciprocal relations with the environments in which they carry out activities of daily living [35-37]. Understanding the complex and dynamic interactions of persons involves the study of the brain’s processes in environmental and social systems. The increasing emphasis of clinical neuropsychology on ecological validity [38,39] and integration with social neuroscience [40,41] is limited by current low-dimensional paper-and-pencil assessments [42]. There is growing attention to the development of high-dimensional tools for assessing and modeling brain functions that include dynamic presentations of environmentally relevant stimuli [36,43,44]. Moving beyond the static or low-dimensional stimuli found in most traditional neuropsychological tests require neuropsychologists to find ways to update their technologies to reflect high-dimensional assessment approaches (eg, deep learning, mobile platforms, wearables, extended reality [XR], and the *Internet of Things* [IoT]).

Neuropsychologists have looked to factor analytic studies of neuropsychological test results to enhance understanding of the functional capacities of patients [45]. However, looking at the relations among responses to low-dimensional tasks that use static or 2D stimuli can constrain task performance and neural activity to abstract constructs (eg, working memory). Low-dimensional assessments bind mean neural population dynamics to a low-dimensional subspace and may limit the neuropsychologist’s assessment of the patient’s ability to perform everyday activities [46]. Furthermore, observation of low-dimensional neural signals may be an artifact of simple cognitive tasks. Standard paper-and-pencil (low-dimensional) tasks often involve basic responses to static or low-dimensional stimuli [47].

Computational neuroscience offers high-dimensional models of cognition via neural network–motivated connectionist models. This approach integrates neuroscience findings into high-dimensional models of the ways in which our brains execute cognitive functions. Leabra is a programing framework that has been used to integrate connectionist models of cognitive function. The result is a holistic architecture adept at producing precise predictions of a broad array of cognitive processes [48,49]. Computational models based on neuroscience findings allow for assessing a model’s sensitivity for capturing a neuropsychological construct and specificity of a given construct to other neuropsychological states and processes. Finally, computational models are shareable and extensible by other neuroscientists who want to extend previous work via iterative construct validation.

### Adoption of Advances in Measurement Science to Neuropsychological Assessment

The NIH Office of Behavioral and Social Sciences Research (OBSSR) also emphasizes advances in measurement science and the move from low-dimensional data analytical approaches (typically linear combinations of features relative to a set of weights for criterion value prediction) to higher-dimensional data analytic approaches for evaluating change over time (eg, deep learning neural networks, machine learning). Clinical scientists are starting to adopt developments in deep learning and other computational modeling approaches [50]. Machine learning and deep learning have been applied successfully in various areas of artificial intelligence research: natural language processing, speech recognition, self-driving cars, and computer vision. For example, natural language processing–oriented computerized neuropsychological assessments have been developed to extract key features of linguistic complexity changes associated with progression in the Alzheimer disease spectrum [51]. High-dimensional data analytics can be applied to computerized adaptive testing (CAT) and computational models derived from large collaborative databases.

High-dimensional measurement protocols offer a clinical scientist with increased precision and granularity of data [10]. Technologically enhanced neuropsychological assessments (including high-dimensional virtual environments [VEs] with graphical models) surpass simple automations (computerized neuropsychological assessments) of low-dimensional paper-and-pencil tasks. Moreover, they allow neuropsychologists to present scenarios that require patients to actively choose among multiple subtasks. From higher-dimensional tasks, context-dependent computational models can be established that include latent context variables that can be extricated using nonlinear modeling.

A framework has been proposed that aims to elucidate probabilistic computations using graphical and statistical models of naturalistic behaviors. The probability distribution for high-dimensional (ecologically valid simulations of everyday activities) tasks is complex. As a result, the brain likely simplifies the high-dimensional stimuli by centering on significant interactions [47]. Neuropsychologists can develop probabilistic graphical models for proficient descriptions of complex statistical distributions that relate several interactions and/or conditional dependencies among neuropsychological variables.

## Deep Learning for Higher-Dimensional Algorithms

Neuropsychologists can use deep learning algorithms that simulate the hierarchical structure of a person’s brain (healthy and damaged). Deep learning is a form of machine learning (ie, algorithms that learn from data to automatically perform tasks such as classification and prediction that can be nonlinear in nature) that processes data from lower dimensionality to increasingly higher dimensions. It is increasingly used to develop novel technologies, big data, and artificial intelligence [52]. Neuropsychologists can use deep learning to analyze studies with both traditional (low-dimensional paper and pencil) and high-dimensional simulation technologies (eg, virtual reality–based neuropsychological assessments, mixed reality, augmented reality). With deep learning, neuropsychologists could process the lower-dimensional data (paper-and-pencil tests). Next, they could move to increasingly higher-dimensional data (eg, from simulation technologies) and develop increasingly complex data-driven semantic concepts that are likely more representative of brain functioning than historical, theoretically based cognitive constructs (eg, working memory).

Probabilistic models and generative neural networks can be used to develop a unified framework for modeling neuropsychological functioning (nonclinical and clinical). Connectionist models such as these are understood to be a portion of the more general framework of probabilistic graphical models. Neuropsychological performances have been modeled as Bayesian computations (brain function expresses perceptions and actions as inferential processes). In this approach, neuropsychological deficits are false inferences arising from aberrant previous beliefs. Bayesian approaches can be used for computational phenotyping that uses graphical models implemented as stochastic processes that involve a randomly determined sequence of observations (each of which is considered as a sample of one element from a probability distribution) via generative neural networks [53]. Visual object recognition (eg, facial processing) can be used as an example. Selective lesions can be applied to computational models of visual object recognition to assess the impact of damage to various cortical regions (eg, early visual processing, extrastriate areas, anterior associative areas). New high-dimensional measures could be developed to assess visual agnosia and examine the appearance of category-specific deficits.

Deep learning architectures can also be used for modeling specific connection pathways in selective impairment. Stochastic decay (stochastic reduction of weight values that decreases responsivity to afferent signals) can be applied to synaptic strengths for examination of cognitive decline. Both global degradation of all network synapses and local degradation of inhibitory synapses from a given processing layer have been investigated. The findings revealed that although older participants accurately performed arithmetical tasks, they had impaired numerosity discrimination on trials requiring the inhibition of incongruent information. They also found that these results were related to poor inhibitory processes measured by standard neuropsychological assessments. The specific degradation of inhibitory processes resulted in a pattern closely resembling older participants’ performance [54]. The addition of computational modeling for the development, validation, and application of neuropsychological assessments represents a high-dimensional approach for neuropsychologists.

## CAT and Item Response Theory

The NIH Toolbox is a battery of computerized neuropsychological assessments that uses item response theory (IRT) and CAT. With IRT, the NIH Toolbox has an alternative to classical test theory as it moves beyond group-specific norms [55]. In IRT, the probability of an item response is modeled according to the respondent’s position on the underlying construct of interest. This approach can be useful for providing item-level properties of each NIH Toolbox measure across the full range of each construct. Although neuropsychological measures tend to meet the reliability and validity requirements of classical test theory, the equivalence of item properties (eg, item difﬁculty and item discriminatory power) is often assumed across items. Consideration of item difﬁculty tends to be subsumed under independent variable manipulation (eg, cognitive load) to modify the marginal likelihood of correct responses in item subgroups. A limitation of this approach is that it does not match well with current item-level analyses found in neuroimaging assessments of brain activations following stimulus probes. For neuropsychological assessments to comport well with brain activation probes, item difficulty needs to be considered to avoid ceiling and ﬂoor effects in patient performances across clinical cohorts. IRT models offer the neuropsychologist both individual patient parameters and individual item characteristics that can be scaled along a shared latent dimension. Neuropsychological assessments would benefit from greater adoption of developments in IRT that emphasize the accuracy of individual items. Various IRT approaches have been applied as signal detection theory models that connect corresponding but discrete methods [56]. Combining IRT and signal detection delivers the measurement accuracy needed for robust modeling of item difficulty and examinee ability.

The NIH Toolbox CAT approach shortens testing time (by about half as long as low-dimensional paper-and-pencil measures). Through avoidance of floor or ceiling effects and concise item pools, CAT delivers equal (or greater) ability–level assessments [57,58]. Moreover, CAT offers enhanced efficiency, flexibility, and precision assessment of multiple domains of interest without adversely affecting participant burden. The application of IRT to CAT provides neuropsychologists with real-time assessment of item-level performance.

## Function-Led Assessments Using High-Dimensional Simulations

Neuropsychologists are increasingly interested in developing assessments that assess the patients’ real-world *functions* in a manner that generalizes to functional performance in everyday activities [38]. A *function-led approach* to neuropsychological assessments involves starting with directly observable everyday behaviors and proceeding backward to observe how a sequence of actions leads to a given behavior. Furthermore, a function-led approach examines how that behavior is disrupted. For example, a patient may have difficulty multitasking while using a global positioning system to navigate a simulated neighborhood in a driving simulator. High-dimensional technologies can be used to present dynamic and interactive stimuli in a 3D environment that includes automatic logging and computational modeling (eg, head movements, eye tracking, response latencies, patterns, etc) of a patient’s performance in everyday activities. High-dimensional neuropsychology tools are being developed and validated to simulate everyday functions (rather than abstract cognitive constructs) [5,41].

Given the drawbacks to experiments conducted in real-life settings (time consuming, require transportation, involve consent from local businesses, costly to use or build physical mock-ups, and difficult to replicate or standardize across settings) and difficulty in maintaining systematic control of real-world stimulus challenges, high-dimensional and function-led XR environments are being used by neuropsychologists.

Low-dimensional (paper-and-pencil and computer automated) neuropsychological tools only indirectly assess the patient’s ability to perform everyday activities [39,59]. VEs offer potential aids in enhancing the dimensionality and ecological validity of neuropsychological assessments through enhanced computational capacities for administration efficiency, stimulus presentation, automated logging of responses, and data analytic processing. Given the precise stimulus presentation and control of dynamic or high-dimensional perceptual stimuli, VEs offer neuropsychological assessments with enhanced ecological validity [5,60-62]. High-dimensional immersive VEs move beyond low-dimensional paper-and-pencil tests with static stimulus presentations in sterile testing rooms to simulated environments that replicate the distractions, stressors, and/or demands found in the real world.

## Data Monitoring With High-Dimensional Technologies

Using passive data monitoring from everyday technologies (eg, smartphones, IoT), clinical scientists can collect real-time cognitive performance throughout the course of a day [10]. Each patient has a digital footprint that transpires from consistent use of everyday technologies. Coupling technologies with developments in measurement science allows for novel approaches to capture cognitions, affects, and behaviors [63]. Rapid progress in sensor technologies has led to objective and effective measures of behavioral performance, psychophysiology, and environmental contexts [64]. For example, machine learning has been employed to extract features from passive monitoring of mobile phone use. When comparing these features with performance on the psychomotor vigilance task, it was found that alertness deviations as small as 11% could be detected [65].

Another example of enhanced data monitoring can be found in the increased granularity in performance assessments and digital logging tools used in the Framingham Heart Study [66]. New developments in digital logging of verbal responses to cognitive stimuli allow for automated algorithms that can extract new language features (eg, speaker turn taking, speaking rate, hesitations, pitch, number of words, vocabulary). These features offer promise for predicting incident cognitive impairment [67]. Furthermore, low-dimensional pencils and pens can be upgraded with high-dimensional digital pens with associated software designed to measure pen positioning 75 times per second. Digital pens have a spatial resolution of ±0.002 inches. For example, digital pens are being used by neuropsychologists for assessing clock drawing performance [68,69]. Minute drawing elements such as pen strokes (eg, clock face, hand, digit) can be logged with greater than 84% accuracy [70]. The sensitivity of these high-dimensional technologies to minute drawing elements, decision-making latencies, and graphomotor characteristics may offer promise to greatly enhance lower-dimensional hand scoring of the Boston Process Approach. A review of the Boston Process Approach and neuropsychology-based technologies has been available recently [71].

## Digital Intervention Platforms

Another transformative opportunity from the NIH OBSSR is the application of high-dimensional technologies to interventions [10]. Progress in neurocognitive rehabilitation has been enhanced by neuroimaging of plasticity of the brain. Similarly, a notable increase can be found in the use of noninvasive brain stimulation approaches that leverage neural plasticity for rehabilitation [72]. Neuropsychologists interested in rehabilitation emphasize the promotion of brain plasticity by increasing a patient’s capacity for performing everyday activities. The resource and labor intensiveness of interventions and the resulting limitations (reach, scalability, and duration) found in real-world assessment environments require interventions to be personalized at the start, adapted throughout treatment, and operationalized into coded databases for fidelity [10].

### Smart Environment Technologies

Smart environments integrate and incorporate several high-dimensional capabilities (eg, function-led evaluation, passive data monitoring, deep learning, etc) to provide both assessment and intervention. Using smart environments, neuropsychologists can discreetly monitor a patient’s everyday activities for changes in clinical status (eg, mobility patterns can predict neurocognitive status). Moreover, automatic interventions can be provided in real-world settings [73-77]. Smart environments use machine learning algorithms (eg, naïve Bayes, Markov, conditional random fields, and dynamic Bayes networks) to model, recognize, and monitor large amounts of labeled training data [78,79]. Activity aware prompting is used to assist in the elevation of independent living. Results from studies using prompting technologies reveal growth in independent activity engagement by patients with neurocognitive impairment [80,81].

### VE Technologies

Smart virtual reality environments simulate real-world scenarios and offer refined stimulus delivery for interventions [60,82,83]. Using VEs, neuropsychologists can present and control stimuli across various sensory modalities (eg, visual, auditory, olfactory, haptic, and kinesthetic). There is an increasing number of validated VEs that can be used for assessment and intervention: virtual apartments [84], grocery stores [85], libraries [86], classrooms [87-90], driving [91], cities [92,93], and military environments [94,95]. In addition to the use of novel measurement science for more efficient assessments using behavioral performances, real-time psychophysiological data (eg, eye gaze) can also be used to adapt assessment and intervention environments for a more individualized approach using factors such as emotional reactivity and ongoing skill development [5,64].

### Smartphones and Other Digital Technologies

Current NIH initiatives for the behavioral and social sciences contend that intervention technologies need to move from short-term assessment and rehabilitation interventions (low-dimensional assessments and treatments that may limit *maintenance* of behavioral response and change) to high-dimensional approaches that use novel technologies (eg, smartphones) to extend treatment duration to improve behavioral maintenance [10]. Mobile technologies offer neuropsychologists higher-dimensional interventions that extend into patients’ everyday activities by logging, monitoring, prompting, and skill building between treatment sessions. One version of this involves ecological momentary assessments and interventions, as patients perform activities of daily living [96-98]. Ecological momentary assessments and interventions using digital devices offer large streams of continuous data [99,100]. Advances in computational modeling offer distinctive prospects for real-time behavioral interventions in ecological contexts [101-104]. As with any new tool, neuropsychologists need to develop and validate measures and interventions.

## Large-Scale Population Cohorts, Data Integration, and Cognitive Ontologies

The NIH OBSSR strategic plan is also interested in big data, data analytics, and data integration techniques for developing collaborative knowledge bases [10]. Integrating neuropsychological data into large collaborative knowledge bases will allow neuropsychologists to either formalize cognitive ontologies or abandon cognitive ontologies for phylogenetically refined functional and neuronal processes that underlie all complex behaviors or more simplistically traditional neuropsychological tasks [8,32,38,105]. Formal designations of distinct sensory, motor, and cognitive entities can be established in terms of parallel, reciprocal, hierarchical, and/or spatiotemporal relations [7,18].

Consistent with critiques from cognitive psychology [8], a limitation of neuropsychological data integration is that low-dimensional neuropsychological assessments are made up of hypothetical interdimensional constructs inferred from research findings [19,38]. Evidence for poor test specificity is apparent in median correlations for common neuropsychological tests. It has been found that although the median correlation within domain groupings on a neuropsychological test was 0.52, the median correlation between groupings was 0.44 [32]. Therefore, the tests are not unambiguously domain specific. The median correlations should be notably higher within groupings and lower between groupings. A recent meta-analysis of relationships between the Wisconsin Card Sorting Test (WCST) and the Weschler Adult Intelligence Scale (WAIS) found a robust relationship between WCST performance and WAIS indices [106]. This is interesting because the WAIS was recently found to be the test most often administered by neuropsychologists and the WCST was the fifth most often administered [2]. Interestingly, the meta-analysis found that WCST scores were associated in comparable strength with both verbal and nonverbal domains from Wechsler Adult Intelligence Scale tests. Another issue is that there is considerable variation in some neuropsychological tests of the same domain (eg, various measures of go or no-go performance) [107]. The shared variance of tests of supposedly differing domains and the lack of consistency in tests of the same domain may decrease the capacity for accurate data integration.

Compounding this issue is the fact that current diagnostic frameworks found in the American Psychiatric Association’s Diagnostic and Statistical Manual of Mental Disorders (DSM) and the World Health Organization’s International Classification of Diseases (ICD) are dependent on presenting signs and symptoms. Moreover, they do not align with findings from genetics and clinical neuroscience [108,109].

Ontologies are formal specifications of entities found in a domain and their relations. An ontology contains designations of separate entities along with a specification of ontological relations among entities with representations via spatiotemporal (eg, *preceded-by* or *contained-within*) or hierarchical relations (eg, *is-a* or *part-of*). This provision of an objective, concise, common, and controlled vocabulary facilitates communication among domains. Neuropsychological assessment lags behind other clinical sciences in the development of formal ontologies [18,19].

As such, neuropsychologists have moved beyond the diagnostic taxonomies found in the DSM and ICD. These diagnostic taxonomies are not sufficient for biomarker research because they do not reflect relevant neurocognitive and behavioral systems. Instead, neuropsychologists interested in developing a common vocabulary for ontologies and collaborative knowledge bases should adopt the US National Institute of Mental Health’s Research Domain Criteria (RDoC) project. The RDoC aims to establish a classification system for mental disorders based on neuroscience and behavioral research [108,110,111].

## Conclusions

Neuropsychologists interested in high-dimensional technologies have embraced the following NIH initiatives to advance scientific developments: (1) integration of neuroscience into behavioral and social sciences, (2) transformative advances in measurement science, (3) digital intervention platforms, and (4) large-scale population cohorts and data integration. Evidence that progress is occurring in neuropsychology exists; however, more work needs to be done. Much of this work involves adoption, development, and validation of novel technologies. Similarly, there is a need for a classification system (based on neuroscience and psychology research) that moves beyond low-dimensional emphases on unitary cognitive constructs specific to a purported functional or neuronal system. A high-dimensional classification instead embraces testable hypotheses of how an observed phenomenon is produced from fundamental underlying mechanisms or processes, the dynamics of those processes (eg, reciprocal, hierarchal, iterative), and the multiple functional or neuronal systems involved in several complex behaviors [8,34]. In more basic terms, neuropsychologists should theorize with verbs instead of nouns to serve scientific progress. Only then can neuropsychologists integrate data to develop meaningful ontologies and collaborative knowledge bases of high-dimensional neuropsychological phenomena. Computational modeling has great promise for achieving this endeavor.

High-dimensional neuropsychology requires substantial reform in the way the profession conducts training. High-dimensional training should be added to current trainings that emphasize primarily (in some programs it may be solely) low-dimensional neuropsychological tests (eg, paper-and-pencil tests) and methods (limited introduction to general linear modeling). Increased emphasis should be placed on technical skill development with high-dimensional technologies and data-driven inferential reasoning. Curricula in neuropsychology programs should be expanded to adapt to the recent technological advances that have led to exponential growth in the other sciences. This would require reimagining training in clinical psychology programs. If neuropsychologists of the future are to work with large collaborative knowledge bases and perform complicated computational modeling of big data, then they need at least basic training in areas traditionally associated with computer science (eg, computer programing) and informatics (algorithms and databases). As such, their basic statistical training would need to be enhanced to include data manipulation, predictive model generation, machine learning, natural language processing, graph theory, and visualization. Increased emphasis on training basic technical and computational skills will improve the ability of future neuropsychologists to participate in science.

A final note is the need for training in neuroethics. Neuroethics has been distinguished into 2 branches: (1) ethics of neuroscience—neuroethics as applied ethical reflection on the practices and technologies found in the neurosciences—and (2) neuroscience of ethics—what neuroscience can reveal about the nature of morality and morally relevant topics [112]. Neuroethics are important for the NIH BRAIN initiative. The NIH BRAIN project aims to examine the ways in which dynamic patterns of neural activity are transformed into cognition, emotion, perception, and action in health and disease [113]. The BRAIN initiative promotes the use of powerful new tools and technologies: (1) technologies for monitoring neural circuit activity and (2) technologies that enable the modulation of neural circuits [114]. As expected, the ethical concerns related to the medical and nonmedical use of neurotechnologies by neuropsychologists are profound. Neuroethics for neurotechnologies include a combination of principlist, deontological, and consequential ethical approaches to answer ethical quandaries [115,116]. Training in neuroethics and the ethical use of high-dimensional technologies will allow neuropsychologists to provide enhanced care for their patients.

## Abbreviations

BRAIN: Brain Research through Advancing Innovative Neurotechnologies
CAT: computerized adaptive testing
DSM: Diagnostic and Statistical Manual of Mental Disorders
ICD: International Classification of Diseases
IoT: Internet of Things
IRT: item response theory
NIH: National Institutes of Health
OBSSR: Office of Behavioral and Social Sciences Research
RDoC: Research Domain Criteria
VE: virtual environment
WAIS: Weschler Adult Intelligence Scale
WCST: Wisconsin Card Sorting Test
XR: extended reality
